# Temperature Simulation of an Ablation Needle for the Prediction of Tissue Necrosis during Liver Ablation

**DOI:** 10.3390/jcm13195853

**Published:** 2024-09-30

**Authors:** Maximilian Will, Thomas Gerlach, Sylvia Saalfeld, Marcel Gutberlet, Daniel Düx, Simon Schröer, Georg Hille, Frank Wacker, Bennet Hensen, Philipp Berg

**Affiliations:** 1 Research Campus STIMULATE, University of Magdeburg, 39106 Magdeburg, Germany; maximilian.will@ovgu.de (M.W.); thomas.gerlach@ovgu.de (T.G.); saalfeldlab@gmail.com (S.S.); gutberlet.marcel@mh-hannover.de (M.G.); duex.daniel@mh-hannover.de (D.D.); schroeer.simon@mh-hannover.de (S.S.); georg.hille@ovgu.de (G.H.); wacker.frank@mh-hannover.de (F.W.); hensen.bennet@mh-hannover.de (B.H.); 2 Department Electromagnetic Compatibility, University of Magdeburg, 39106 Magdeburg, Germany; 3 Department for Medical Informatics, University of Kiel, 24118 Kiel, Germany; 4 Institute of Diagnostic and Interventional Radiology, Hanover Medical School, 30625 Hanover, Germany; 5 Department of Simulation and Graphics, University of Magdeburg, 39106 Magdeburg, Germany; 6 Department of Medical Engineering, University of Magdeburg, 39106 Magdeburg, Germany

**Keywords:** microwave ablation, computational fluid dynamics, MRI thermometry, tumor ablation

## Abstract

**Background/Objectives:** Microwave ablation (MWA) is the leading therapy method for treating patients with liver cancer. MWA simulation is used to further improve the therapy and to help develop new devices. **Methods:** A water-cooled ablation needle was reconstructed. MWA simulations of a polyacrylamide phantom were carried out and compared with a representative clinical example (tumor diameter: 8.75 mm). The Arrhenius damage model and a critical temperature approach of 60 °C were applied to assess the necrosis zones. Finally, the simulation results were compared to the corresponding MR measurements. **Results:** Most of the heating in the simulation took place at a distance of 5 mm along the transverse axis and 20 mm along the longitudinal axis above the needle tip. The calculated Dice scores for the Arrhenius model were 0.77/0.53 for the phantom/clinical case. For the critical temperature approach, Dice scores of 0.60/0.66 for the phantom/clinical case were achieved. **Conclusions:** The comparison between simulated and measured temperature increases showed an excellent agreement. However, differences in the predicted necrosis volume might be caused by omitting consideration of the heat sink effect, especially in the clinical case. Nevertheless, this workflow enables short MWA simulation times (approximately 3 min) and demonstrates a step towards possible integration into daily clinical use.

## 1. Introduction

Liver cancer is not only common worldwide, but it is also one of the leading causes of death in cancer patients [1,2]. Accordingly, there is an urgent need to advance existing treatment methods and develop new methods to improve therapy outcomes.

Microwave ablation (MWA) is a minimally invasive approach and has emerged as a safe and efficient therapy method for liver tumors [3,4]. This heat-based technique is used to destroy tumor cells locally while the surrounding tissue can be mostly preserved. Generally, MWA simulation can improve outcomes further and help develop more comprehensive therapy devices [5]. Several computational models for clinical planning and predicting the ablation procedures as well as the treatment outcome have already been developed [6].

Singh and Melnik coupled a thermo-electro-mechanical model that accounts for heat relaxation times; however, they focused exclusively on a 2D approach [7]. Gao et al. [8] presented a novel characterization of and mapping method for thermal coagulation zones based on the finite element technique, but it lacks clinically applicable simulation and analysis times. In addition to these studies, previous works focused on specific subtopics, e.g., tissue shrinkage or the heat sink effect and their impact on MWA treatment [9,10] or investigating evaporation at temperatures above 100 °C [9,10,11]. Furthermore, damage models were used to analyze thermally induced cell damage [12]. Here, the Arrhenius model, the critical temperature, and the CEM43 model are commonly applied. These enable predictions about which volumes are necrotic [13,14].

The accuracy of a microwave ablation simulation hinges on three key factors: the precise modeling of the electric field generated by the applicator, the accurate representation of tissue properties, and the detailed modeling of the anatomical structures involved. The precise design of applicators used in commercial microwave generators is often proprietary information. Consequently, phantom experiments, with their homogeneous materials and simple configurations, provide a valuable platform for validating the accuracy of our microwave applicator models. Unlike clinical data, which typically only provides the final size of the ablation zone, phantom experiments allow for a direct comparison between simulated and measured temperature profiles by incorporating temperature sensors. The clinical utility of the simulation can only be assessed through clinical cases. This study aimed to model and validate the electric field of a commercial microwave applicator in phantoms, and to evaluate the simulation’s accuracy in a patient, considering additional influencing factors.

## 2. Materials and Methods

In the following subsections, the technical methodology of the three main focus areas is described. This comprises (I) MR-based in vitro phantom measurements, (II) the detailed computational MWA modeling and (III) the clinical ablation considering a comprehensive real case.

### 2.1. Magnetic Resonance Image-Guided Microwave Ablation Experimental Setup

**Figure 1:**
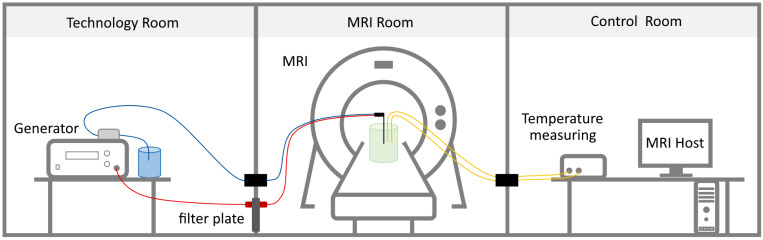
Experimental setup of in vitro MR-guided MWA [15].

The experiments were performed on a 1.5 T MAGNETOM Avanto (Siemens Healthcare, Erlangen, Germany) magnetic resonance imaging (MRI) device. The ECO-100E microwave generator (Eco Microwave System Co., Ltd., Nanjing, China), referred to as *ECO* in this paper was used to perform the thermal ablations. The generator operates at a frequency of approximately 2.45 GHz and with a maximum power of 150 W. It can be operated in continuous and pulsed modes. The experiments for this research were carried out exclusively in continuous mode. The generator has an active water cooling system for cooling the applicator, which uses a roller pump. For this purpose, the applicator has two connections through which the water is fed in and out. The applicator and the generator are connected via a coaxial cable (8 m length) to perform the MWA. The power losses occurring through the cables were gathered from an information sheet and later used for the simulations. The applicator has a diameter of 15 Gauge (1.45 mm) and a length of 150 mm. To determine the temperature during ablation, two fiber-optic temperature sensors (FOTEMPTrafo, Weidmann Technologies Deutschland GmbH, Dresden, Germany) were used. A schematic representation of the experimental setup can be seen in Figure 1.

### 2.2. Polyacrylamide Liver Phantoms

The ablations were performed in phantoms made out of polyacrylamide and bovine serum albumin according to Bu-Lin et al. [16]. The phantoms are designed to mimic the physical properties of liver tissue, such as density, electrical conductivity, specific heat capacity and coagulation temperature. The coagulation temperature and the electrical conductivity of the phantom are dependent on the pH value (4.2–4.8) [16,17]. The mean values of the pH-dependent parameters were used to simulate the phantom. At room temperature (22 °C), the phantoms are transparent. During coagulation, the phantoms turn white. The phantoms were prepared in cylindrical plastic tubes with an inner diameter of 102.9 mm. These tubes were filled up to a height of about 125 mm.

### 2.3. MR Measurements

**Figure 2:**
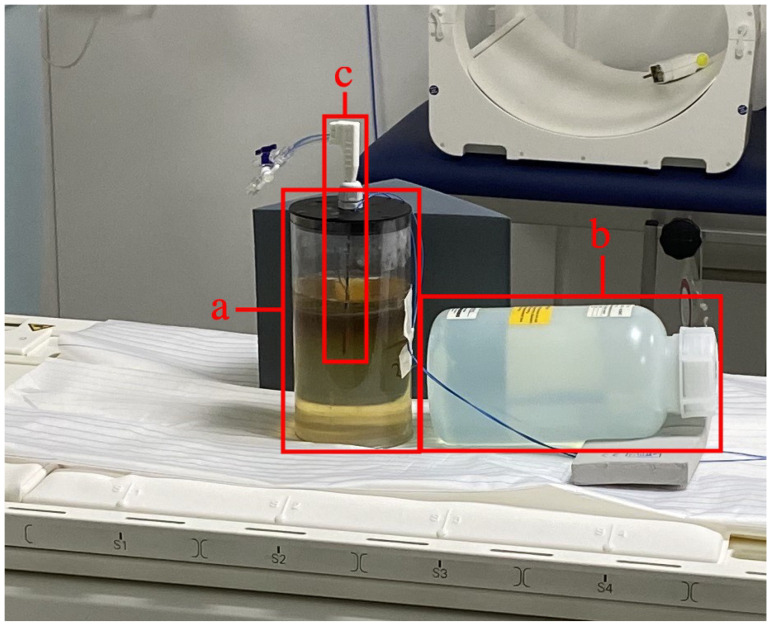
Experimental setup of the liver phantom: (**a**) liver phantom, (**b**) cooling water reservoir, (**c**) ECO ablation needle [15].

To enable MRI with as little electromagnetic interference as possible, the MWA generator was set up in the MRI’s technical room. Water cooling for the applicator was provided by the generator’s roller pump and a small water reservoir. The water hoses were fed into the MRI room via a small pipe (wave guide). The experimental setup for the phantom can be seen in Figure 2. The generator was connected to a filter plate via a coaxial cable. On the other side of the filter plate, the coaxial cable for MWA was connected [18]. The phantom for the application of MWA was set up on the MRI table. The applicator was placed in the center of the phantom. Two temperature sensors were inserted vertical and parallel to the applicator at a distance of 1 cm and 2 cm and 1.8 cm above the needle tip. The placement of the temperature sensors is described in Section 2.7. For the MRI, a multi-channel receiver coil was placed on top of the phantom.

The fiber-optic cables of the temperature sensors were fed into a control room through a second wave guide, where they were connected to a measuring device.

**Figure 3:**
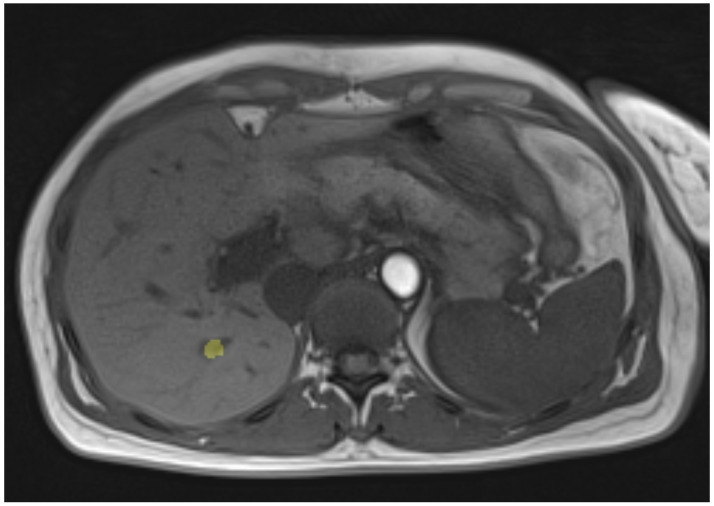
Pre-ablation image of the patient. Note that the tumor area is marked in yellow.

**Figure 4:**
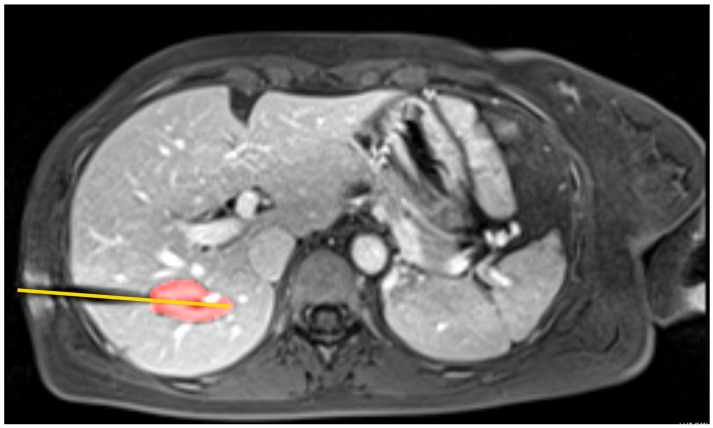
Post-ablation image with the ablation needle from the clinical case, which was later used for simulation. Note that the yellow line shows the needle trajectory and the red area shows the approximate post-ablation zone.

The patient was treated using intraoperative MRI (iMRI). A liver tumor was treated with an ablation time of approximately 7 min. The tumor was approximately 11 mm long and 8.7 mm wide. There were no additional temperature sensors used in the clinical case. The post-treatment MRI data was extracted by a physician and serves as the basis for comparison with the simulation of the patient case. Exemplary MR images of the pre- and post-interventional clinical case can be seen in Figure 3 and Figure 4, respectively. The study was conducted according to the guidelines of the Declaration of Helsinki and approved by the Ethics Committee of University Hospital Hanover (protocol code 3227-2016/October 2016).

### 2.4. Computational Modeling

The computational time was between 45 and 63 min (Intel Core i5-8600k, 16GB RAM, Intel Corporation, Santa Clara, CA, USA). The simulations were implemented using the finite element method in COMSOL Multiphysics v.5.6 (COMSOL AB, Stockholm, Sweden). A simulation of the phantom for a duration of 10 min was carried out. The electromagnetic field propagating from the needle is solved in the first, frequency-dependent step of the simulation. It is then used to perform the second, time-dependent part of the simulation, where the heat propagation is calculated by applying the modules for electromagnetic processes and fluid and heat propagation. The electromagnetic frequency domain formula implemented in COMSOL calculates the propagation of electromagnetic waves and the related power absorption in the tissue. This is coupled with the integrated bioheat and linear heat transfer model of COMSOL in order to calculate the temperature profiles of the needle in the relevant domains of the phantom and liver tissue. Specifically, Pennes’ bioheat equation is used to calculate heat transfer in biological tissue and vasculature [19]:

(1) $$\rho_{T}C_{T}\frac{\partial T}{\partial t}=k_{T}\nabla^{2}T+\rho_{b}C_{b}\omega_{b}\left(T_{b}-T\right)+Q_{m}+Q_{ext}+Q_{E}$$

where $\rho_{T}$ is the tissue density, $\rho_{b}$ the blood density (kg/m^3^), $C_{T}$ and $C_{b}$ are specific heat capacity (J/(kg·K)) of tissue and blood, respectively, $k_{T}$ is the tissue thermal conductivity coefficient (W/(m·K)) and $Q_{m}$ (W/m^3^) the heat generated by metabolism, which is small in comparison to the heat generated by the MWA itself. The external heat source $Q_{ext}=\frac{{\sigma |E|}^{2}}{2}$, equals the heat produced by the dielectric heating of the electromagnetic field, where σ is the electrical conductivity of the tissue (S/m) and E the electrical field (V/m). The vaporization energy $Q_{E}$ is the energy needed to vaporize tissue water. The thermal influence of blood perfusion $\omega_{b}$ (1/s) and the metabolic heat $Q_{m}$ (W/m^3^) was neglected for the phantom case. The value for the metabolic heat in the patient case can be looked up in Table 1. The vaporization energy $Q_{E}$ (W/m^3^) was not considered in the simulations.

### 2.5. Modeling the MWA Needle

Similarly to other studies, a water-cooled ablation needle was simulated [12,20,21]. This was conducted by modeling the monopole coaxial needles from ECO. The needle consists of an inner and outer copper conductor. It is coated with a dielectric material, presumably PTFE (polytetrafluoroethylene). The needle tip consists of a ceramic/plastic component which is not visible during MR. The microwave antenna is located approximately 25 mm above the tip of the needle and the inner conductor is additionally coated with another plastic dielectric. The microwaves are emitted in the gap between the inner conductor, the dielectric and the tip. For the cooling water circulating in the needle, a simplification was used. A connective heat transfer boundary, with a constant cooling temperature of approximately 7 °C for the phantom and 20 °C for the clinical case were used. Changes in the tissue parameters due to the influence of temperature increases were neglected. The parameters used for the different materials as well as the tumor tissue, such as relative dielectric, electric conductivity, relative permeability, density and specific heat capacity, were taken from the literature [14,20,22,23]. The geometry of the tumor detected by MRI prior to ablation was used to recreate the dimensions of the tumor in the simulation for the clinical case. The parameters used for both simulations are summarized in Table 1.

Two important parameters for MWA simulations are electrical conductivity and dielectric permittivity [5]. These differ both for the tissue and for the frequency used. Common frequencies used in MWA systems are 915 MHz and 2.45 GHz [5,24].

**Figure 5:**
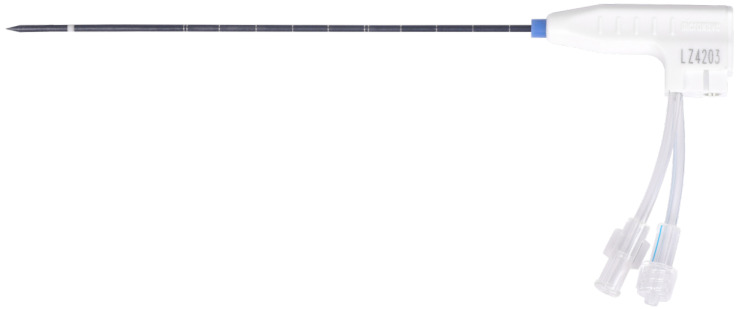
Display of the monopole coaxial applicator model from ECO used for both phantom experiments as well as treating the patient [25].

The ECO needle used in the experiment was operated with a generator power of 80 W and at a frequency of 2.45 GHz (see Figure 5). After power losses due to the cables (8 m length) from the experimental setup, approximately 35 W arrive at the ablation needle and were used for the simulation. For the clinical case, however, a ECO 200 generator with an input power of 120 W was used. The exact power loss is not known but was estimated using a data sheet from the developer to be at approximately 60–70%.

**Table 1:** Simulation parameters used for simulating liver phantoms and the clinical case [14,20,22,23].

Name			
Variables	Value	Unit	Description
$\rho_{blood}$	1000	kg/m^3^	Density, blood
$Cp_{blood}$	3639	J/(kg·K)	Specific heat, blood
$\omega_{blood}$	0.00361	1/s	Perfusion rate, blood
$T_{blood}$	310.15	K	Temperature, blood
$T_{phantom}$	290.15	K	Temperature, phantom
$\epsilon_{liver}$	43.03	1	Relative permittivity, liver
$\epsilon_{tumor}$	54.8	1	Relative permittivity, tumor
$\sigma_{liver}$	1.69	S/m	Electric conductivity, liver
$\sigma_{tumor}$	2	S/m	Electric conductivity, tumor
$\epsilon_{diel}$	2.03	1	Relative permittivity, dielectric
$\epsilon_{cat}$	2.6	1	Relative permittivity, catheter
*f*	2.45	GHz	Microwave frequency
$P_{in}$	35	W	Input microwave power phantom
$P_{inP}$	42	W	Input microwave power clinical case
$Cp_{liver}$	3600	J/(kg·K)	Specific heat capacity, liver
$Cp_{tumor}$	3760	J/(kg·K)	Specific heat capacity, tumor
$\rho_{liver}$	1069	kg/m^3^	Density, liver
$\rho_{tumor}$	1040	kg/m^3^	Density, tumor
$k_{liver}$	0.55	W/(m·K)	Thermal conductivity, liver
$k_{tumor}$	0.57	W/(m·K)	Thermal conductivity, tumor
$Q_{mliver}$	1000	W/m^3^	Metabolic heat, liver
$Q_{mtumor}$	1.3 × 1000	W/m^3^	Metabolic heat, tumor

Due to the simulation of liver tissue, the metabolic heat coefficient $Q_{m}$ from Pennes’ bioheat Equation (1) was assigned to both liver and tumorous tissue. A 120 W microwave generator was used to treat the patient and, due to power losses, approximately 42 W were emitted at the needle.

### 2.6. Discretization

**Figure 6:**
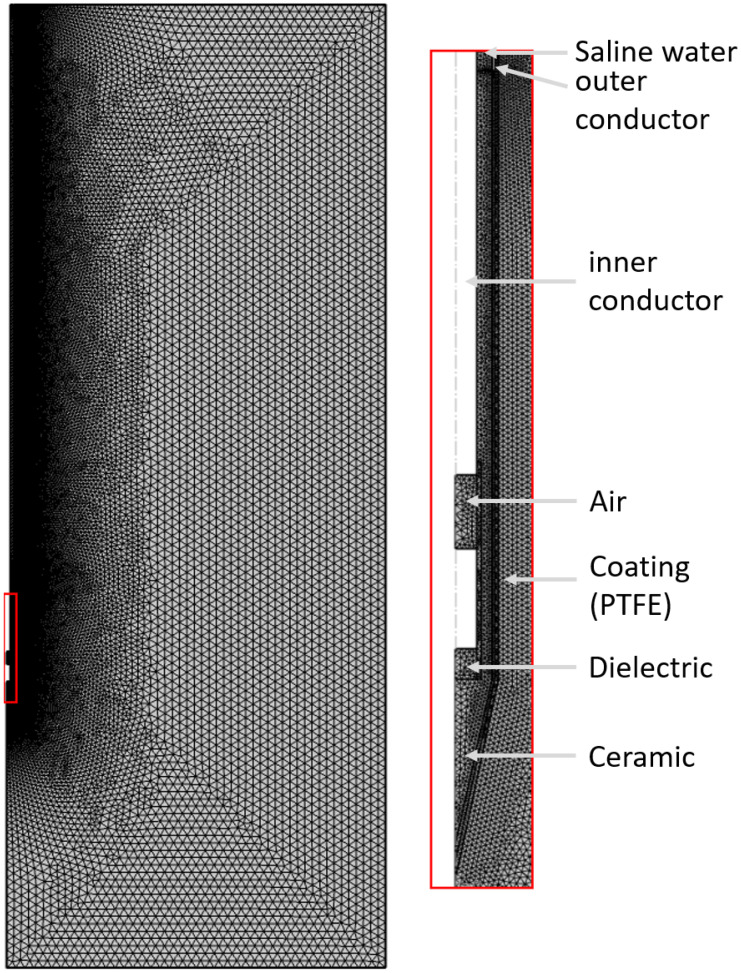
Physics-controlled simulation mesh. On the right side is a magnified view of the needle tip. Note that the mesh is much finer around the needle area (down to 0.0025 mm).

For the spatial discretization of the domain considered, an unstructured tetrahedral mesh for the whole geometry (phantom, liver tissue, tumor tissue and needle) was created in COMSOL. The mesh was manually refined in the region of interest (close proximity to the needle). The smallest cell sizes, 0.05 mm, were found in the vicinity of the ablation needle, increasing outwards to a maximum cell size of 3 mm. A mesh study was performed to ensure that the accuracy of the simulation was not affected. The simulation mesh consisting of 62832 tetrahedral elements can be seen in Figure 6. The simulation times were set to 10 min in order to correspond to the experiments.

### 2.7. Analytic Approach

The Arrhenius model is a well-established model for predicting cell damage induced by thermal ablation [12,26]. It is used to calculate the cell damage generated in the simulation then compare it with data from the previously acquired results from phantom and patient measurements, which are based on image contrast: T2-weighted in the phantom and post-ablative T1-weighted after a contrast agent was given for the clinical cases. The following equation from the Arrhenius model implemented in COMSOL was used to calculate thermal damage:

(2) $$\Omega \left(T\right)=\int_{t=0}^{t}Ae^{-\frac{E_{a}}{R(T(t)+273.15)}}dt$$

The frequency factor ($A=7.39\times 10^{39}$ L/s) and the activation energy ($E_{a}=2.577\times 10^{5}$ J/mol) were automatically selected from COMSOL according to the material type “liver tissue” [27]. R is the universal gas constant (8.3145 (J/(mol·K))), and T(t) is the temperature (°C) at time t. The binary thermal damage map was calculated using I=Ω(T)>1, meaning 63% of the thermal damage process was completed [12].

**Figure 7:**
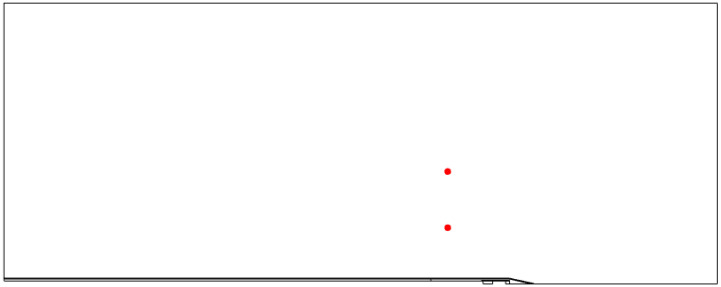
Measuring points (red) for the temperature created in COMSOL to compare with the data from the phantom experiment.

In addition, 3D plots of the ablation zones were created using a 360° rotation of the 2D axisymmetric display of the Arrhenius damage model as well as the 60 °C temperature isoline [11]. These are based on the Arrhenius model and can be exported as 3D objects for further quantification. The Dice similarity coefficient (Dice score), Euclidean distance and Hausdorff distance were calculated using Matlab (version: 9.13.0 (R2022b), Natick, MA, USA: The MathWorks Inc.; 2022). These distance metrics were used for the quantitative analysis [28,29]. The ablation volume from the clinical case was segmented on T1 post-contrast imaging by a radiologist with 2 years of experience. The registration of the volumes was carried out by a transformation matrix, which was calculated by a previous manual surface registration of both volumes in the open-source mesh processing tool MeshLab (MeshLab Development Team, Version 2022.02, Pisa, Italy). Subsequently, the registration was implemented in Matlab, and the distance values were calculated. The procedure used by MeshLab for registration and Matlab for calculation of the distance metrics was identical. Post-ablative imaging to determine the ablation zone was registered with the Advanced Normalization Toolbox to pre-ablative imaging to determine the location and size of the tumor to compensate for respiratory motion [30]. For the simulation, several measuring points were set at a distance of 5 mm and at a height of 20 mm above the needle tip to evaluate the temperatures reached in the phantom case and are displayed in Figure 7. The temperature over time can be observed at these points during the simulation.

## 3. Results

### 3.1. In Vitro Phantom

**Figure 8:**
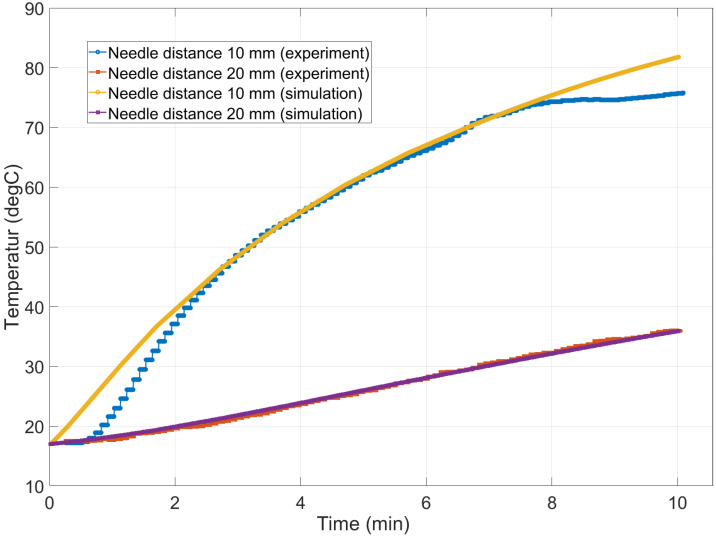
Comparison of the time-dependent temperature changes during a 10 min simulation of the liver phantom with the temperature sensors from the experiments. The measurements were recorded approximately 18 mm above the needle tip.

**Figure 9:**
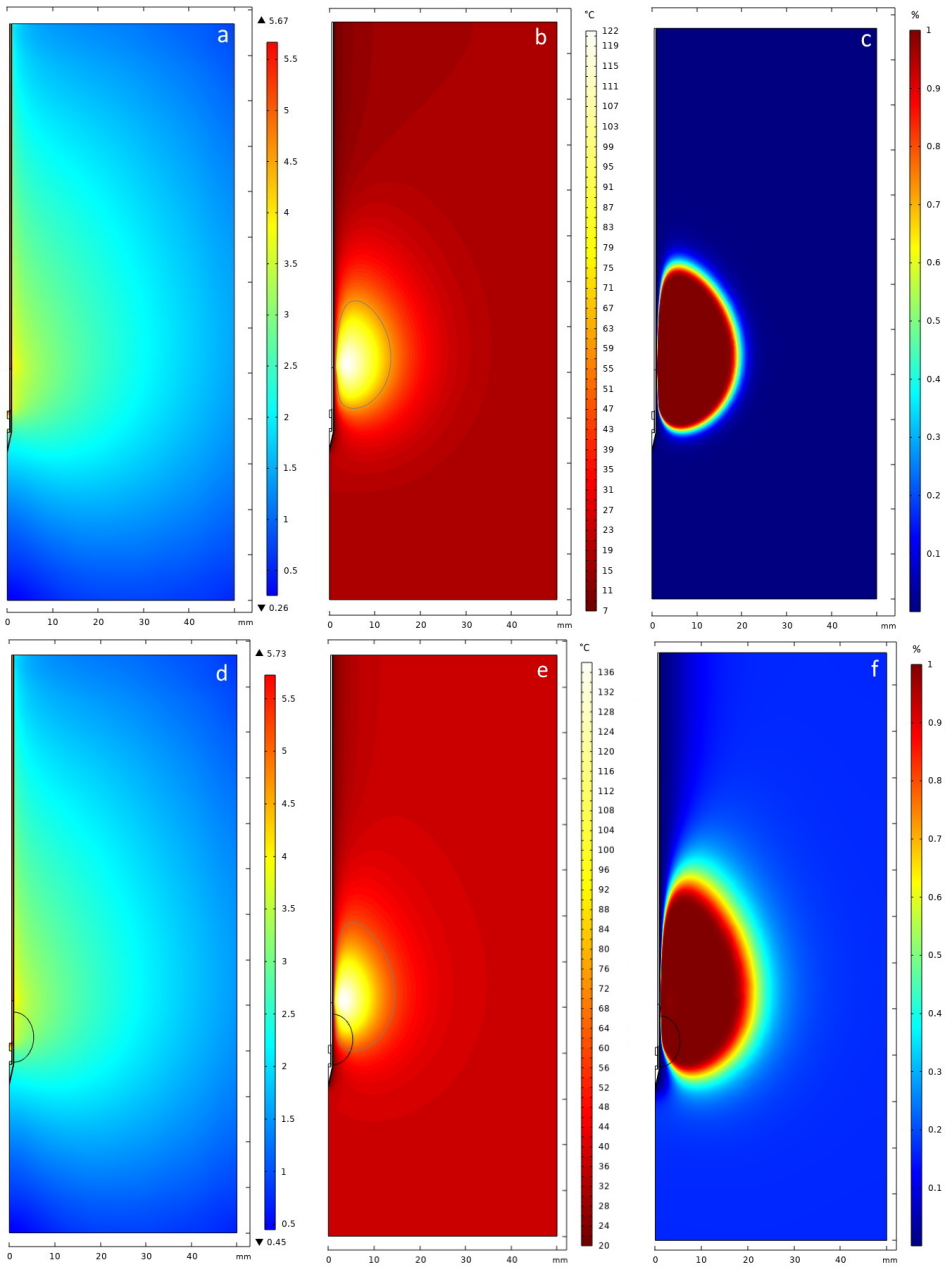
Qualitative evaluation of the simulation results after 10 min, where (**a**–**c**) represents the phantom and (**d**–**f**) the clinical case (the black contour represents the tumor). (**a**,**d**) The electrical field was calculated with an input power of 35 W for the phantom and 42 W for the clinical case, respectively. The electric al field in COMSOL is displayed using a logarithmic scale; (**b**,**e**) 2D temperature field. The gray line marks a 60 °C isoline; (**c**,**f**) 2D Arrhenius kinetics provides the degree of tissue injury from 0 to 100% (the threshold used was 98%).

The temperature development during the ablation process is shown in Figure 8. The temperatures reached during the simulation were 115.14 °C and 122.2 °C at 7 min and 10 min, respectively. The temperature increases most significantly within the first third of the procedure, marked by a sharp rise, which is particularly noticeable 10 mm along the transverse axis of the needle. Beyond this initial phase, the increase in temperature becomes more gradual and steadier. Nevertheless, the maximum temperature for the simulation is not reached after 10 min, which can be recognized by the relatively steep rise in the temperature curve. The areas with the strongest heating and therefore probably also with the highest energy input were observed at a distance of approximately 4 mm from the needle. The temperature propagation in the 2D axisymmetric simulation environment was plotted. It is noticeable that most of the heating occurs at a height approximately 20 mm above the tip of the needle. There is no direct heating of the tissue at and above the needle tip. In addition, the tissue damage calculated by the Arrhenius model is displayed next to the 2D temperature. The area output by the Arrhenius model is larger than that of the 60 °C isoline. The qualitative results for the liver phantom can be seen in Figure 9.

The Arrhenius model shows moderate agreement with the results from the experiment in all distance metrics and with a Dice score of 0.77. The simulated ablation zones are smaller in comparison to the segmented one from the experiments. The values from the 60 °C temperature approach shows more deviation from the experimental results. This is also highlighted by the lower Dice score of 0.60. The values for the distance metrics are displayed in Table 2.

### 3.2. Clinical Case

Similarly to the phantom simulation, the clinical case was simulated for 10 min. The overall highest temperatures reached after 7 min and 10 min were 133.71 °C and 135.52 °C, respectively. The electrical field, 2D temperature distribution as well as the tissue damage calculated by the Arrhenius model for the clinical case are displayed in Figure 9.

Due to a higher input power, the electrical field has a higher magnitude, although the distribution remains similar. The temperature distribution as well as the Arrhenius tissue damage differ from the phantom results. Especially for the Arrhenius model, more scattering occurs and the boundary between preserved and necrotic tissue is more diffuse. It is noticeable that the tumor border is not fully covered in both the 60 °C isoline as well as the Arrhenius damage Figure 9. The same distance metrics were used for the quantitative evaluation as in the simulation of the phantom. The results can be seen in Table 3.

**Figure 10:**
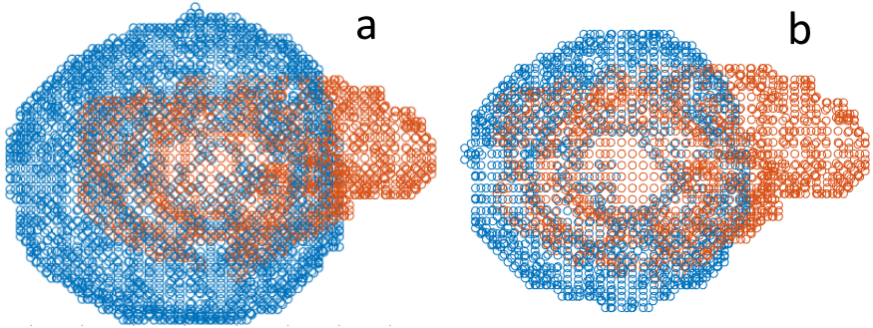
Registered pointclouds of the two ablation zones (blue) from the (**a**) Arrhenius and (**b**) 60 °C temperature approaches from the clinical case. The segmented ablation zone from the clinical case is colored in orange. Note that (**b**) fits the size of the segmented ablation zone much better.

At 9.86 mm, the Hausdorff distance for the Arrhenius approach shows similar results when compared to the phantom case. The Euclidean distance and the Dice score for the Arrhenius model show better agreement compared to the simulation of the phantom. The results of the 60 °C approach appear to be better suited, with a Dice score of 0.66. Compared to the phantom case, the size of the simulated ablation zone is closer to the patient’s segmented ablation zone. This can be also observed in Figure 10 where the registered point clouds of the patient case are shown for both damage models.

## 4. Discussion

MWA is an established method for the treatment of liver cancer. In addition, image-based numerical simulation has proven to be an important component for investigating the MWA process as well as helping to further improve this therapy method [10,31,32]. In this study, the heat propagation of an ECO MWA needle was investigated using simulations as well as experimental results from a liver phantom and a real clinical case. A major focus was on modeling the water-cooled ECO needle using angiographic images as well as reducing computational effort to maintain the clinical usability of the simulation results, unlike complex 3D simulations that require computer clusters for computation [14,33]. The axial symmetry of the needle and the simple phantom geometry material composition were used to create a simulation with minimal computational effort, in contrast to the work of Radjenovic et al. [11] and Heshmat et al. [34], where complex 3D models were used, which have to be processed for each individual case and are very computationally expensive. In addition, high-quality imaging using MRI or even CT are a burden on the patient, which should not be neglected in therapy planning. In addition, the accuracy of the microwave applicator model was assessed through a phantom experiment.

### 4.1. Implementation of the Simulation

The temperatures reached during the simulation of the phantom as well as the patient case are realistic but in some areas higher than 100 °C. This leads to the vaporization of water and the carbonization of tissue, which can lead to worse therapy outcomes and damage to healthy tissue [9,35]. For that reason, temperatures over 100 °C are mostly avoided in practice. This can be performed through the design of the MW antenna, use of pulsed mode and by cooling the microwave applicator [36,37].

However, good agreement between the experiments and simulation can be seen in Figure 8. Here, the time-dependent temperature curves seem to show good agreement between the simulation and experiments, which is consistent with the findings of Tehrani et al. [38]. The maximum temperature for the clinical case is reached much faster compared to the phantom case. An important factor is the higher starting temperature of 37 °C for the patient case compared to 17 °C for the phantom, as well as a higher power input of 42 W. The simulation produces smaller ablation zones compared to the experiments, with a Dice score of 0.526 for the Arrhenius model. For the phantom case, the simulation seems to underestimate the volume of ablated tissue. In contrast, the size of the ablation zone is close to the patient’s segmented ablation zone from the clinical case. However, problems occur when it comes to shape and appearance. This is in agreement with the statements of Prakash [33], where the 2D approach is not sufficient for clinical application, because of the much more heterogeneous geometry of real liver tissue. Normally, in a phantom case, a MWA needle produces a more or less spherical or tear-shaped ablation zone, where tissue damage starts out from the tip of the needle. This is also shown in the work of Yang et al. [39], who carried out similar experiments with an ECO needle array. However, this spherical ablation did not occur at the needle tip as one might expect. The area directly longitudinal to the needle tip shows no signs of ablated tissue. In contrast, most of the heating takes place approximately 20 mm orthogonally in the opposite direction to the needle tip. This is probably due to the reconstructed needle geometry, which does not completely replicate the actual needle. Further investigation is required and changes should be applied to improve the realism of the simulation. It is difficult to compare the heating profiles of different needles and needle types, but the results from other studies show qualitatively similar temperature curves over time [11,23,35].

However, the MRI images of the post-ablation zone also show a non-spherical shape, which can be seen in Figure 10. The non-spherical shape of the ablation zone depends on several factors. The heat sink effect usually occurs more noticeably in the radio frequency but is less observed in MWA. The heat sink effect also plays a role if the tumor is located near larger blood vessels, which was the case with this patient [10,21]. The 2D axisymmetric simulation of the phantom can reproduce the qualitative ablation zone but not its size. In contrast, larger and relevant deviations occur in the clinical case. Nevertheless, the modeled ablation needle has been shown to be functional and capable of creating ablation zones of realistic sizes. The construction as well as the successful simulation of an ablation needle comes with a lot of hurdles, which have been successfully met to a large extent in this work [40,41]. A correction to the geometry (e.g., a change in material thickness and the structure of the needle) could improve future simulation results and is necessary and would change the power output and distribution of the electrical field produced by the needle. This requires more detailed knowledge of the structure of and materials in the needle as well as the parameters of the ablated tissue, particularly for tumorous tissue.

### 4.2. Limitations

**Figure 11:**
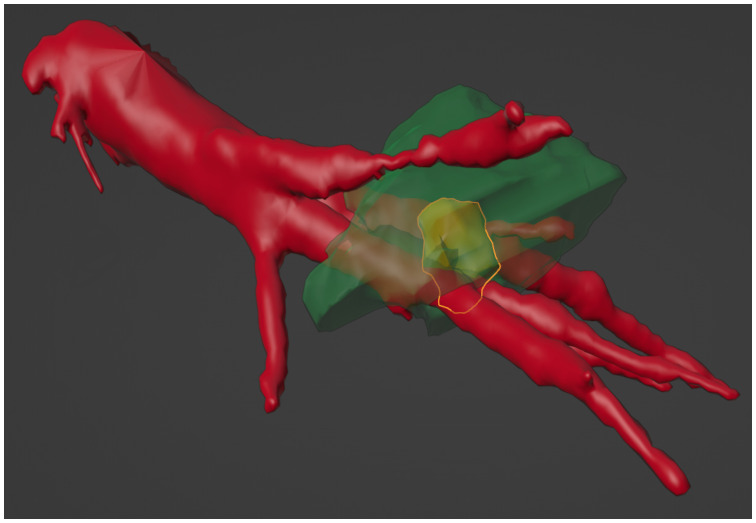
Segmented 3D ablation zone as well as the target surface from pre-ablation planning (yellow) with vessels from the clinical case. Note that the vessel (red) pierces through the ablation zone (green), which requires consideration of the heat sink effect.

It is important to state that a simulation always involves compromises. The human body is far more complex than a phantom as well as in need of different simulation parameters. Phenomena such as the heat sink effect and vaporization of water in the tissue were neglected in this research. Other studies have implemented those aspects, but this involves more computational effort, where a short computation time was an important factor here [11,38]. However, the clinical case showed that it is definitely important to consider those factors. The MRI images of the patient show that the tumor is located next to a large blood vessel. Its effect on the MWA treatment can also be seen in the post-ablation images as well as in the literature [10,42]. A hole can be found in the segmented ablation zone, which highlights the impact of the vessel on therapy planning. The segmented ablation zone is shown in Figure 11. Another limitation is the simplified cooling of the needle as a boundary condition. Temperature-independent parameters should be used in future research. Some of the deviations can also be explained by the registration process. Errors inevitably occur during the manual determination of markers while performing the registration. The rotation of the 2D axisymmetric ablation zones and refinement methods from the simulations are also subject to small errors, as these only represent an approximation of the real 3D ablated tissue and depend on the mesh size used for the simulation. Although a functional ablation needle could be reconstructed, some questions are raised. The position of the highest temperature region is not consistent with observations from everyday clinical practice. The reason for this must lie in the needle geometry. Future investigations may lead to new insights regarding that problem. The same applies to the significantly smaller ablation zones for the phantom case, with well-matching temperature curves. A deviation in the placement of the temperature sensors in the phantom as well as insufficient power used on the needle could introduce errors.

## 5. Conclusions

In this study, a functioning ablation needle was reconstructed and modeled using angiography images from an ECO ablation system. With the large vessel directly next to the tumor, a rather difficult case was deliberately chosen for the simulation. In this respect, the difficulty of developing a generally good simulation is demonstrated. The 2D axisymmetric approach allowed for very short computational times of only 3 to 5 min. The Arrhenius damage model and a critical temperature model of 60 °C were used to evaluate the ablation zones. The simulation results were compared with segmented ablation zones from an experimental phantom and a clinical case. Good results regarding the temperature curves were obtained. For the clinical case, the simulation lacks additional parameters and consideration of the heat sink effect, which seemed very prominent in the clinical case presented. Even though the reconstructed needle could be modeled in detail, it has to undergo changes to further improve its performance in creating realistic ablation patterns.

## Figures and Tables

**Table 2:** Distance metrics of the simulation damage volumes and segmented ablation zone from the liver phantom.

Metric	Arrhenius	Critical Temp. 60 °C
Hausdorff D.	12.021 mm	15.017 mm
Euclidean D.	2.504 mm	3.754 mm
Dice score	0.772	0.601

**Table 3:** Distance metrics of the simulation damage volumes and segmented ablation zone from the patient geometry.

Metric	Arrhenius	Critical Temp. 60 °C
Hausdorff D.	9.862 mm	11.045 mm
Euclidean D.	3.061 mm	2.502 mm
Dice score	0.526	0.663

## Data Availability

Due to the large amount of data along the complex workflows of experimental and numerical temperature acquisitions, the specific raw data supporting the conclusions of this article will be made available by the authors on request.

## Abbreviations

The following abbreviations are used in this manuscript:

MWA	Microwave ablation
MRI	Magnetic resonance imaging
